# Cancer associated fibroblasts confer shear resistance to circulating tumor cells during prostate cancer metastatic progression

**DOI:** 10.18632/oncotarget.27510

**Published:** 2020-03-24

**Authors:** Nerymar Ortiz-Otero, Andrea B. Clinch, Jacob Hope, Wenjun Wang, Cynthia A. Reinhart-King, Michael R. King

**Affiliations:** ^1^ Meinig School of Biomedical Engineering, Cornell University, Ithaca, NY 14850, USA; ^2^ Department of Biomedical Engineering, Vanderbilt University, Nashville, TN 37202, USA

**Keywords:** metastasis, cancer associated fibroblasts, circulating tumor cells, cytoprotection, collective migration

## Abstract

Previous studies have demonstrated that CTCs do not travel in the bloodstream alone, but rather are accompanied by clusters of stromal cells such as cancer associated fibroblasts (CAFs). Our laboratory has confirmed the presence of CAFs in the peripheral blood of prostate cancer (PC) patients. The observation that CAFs disseminate with CTCs prompts the examination of the role of CAFs in CTC survival under physiological shear stress during the dissemination process using a clinically relevant, three-dimensional (3D) co-culture model. In this study, we found that “reactive CAFs” induce shear resistance to prostate tumor cells via intercellular contact and soluble derived factors. In addition, these reactive CAFs conserve the proliferative capability of tumor cells in the presence of high magnitude fluid shear stress (FSS). This reactive CAF phenotype emerges from normal fibroblasts (NF), which take on the CAF phenotype when co-cultured with tumor cells. The reactive CAFs showed higher expression of α-smooth muscle actin (α-SMA) and fibroblast activation protein (FAP) compared to differentiated CAFs, when co-cultured with PC cells at the same experimental conditions. Together, we found that the activation mechanism of NF to CAF comprises different stages that progress from a reactive to quiescent cellular state in which these two states are differentiated by the fluctuation of intensity in CAF markers. Here we determined that a reactive state of CAFs proved to be important for supporting tumor cell survival and proliferation. These findings suggest the use of CAFs as a marker for cancer progression and a potential target for novel cancer therapeutics to treat metastatic disease.

## INTRODUCTION

Prostate cancer (PC) is the second leading cause of cancer related death in western countries, with most of these deaths attributed to cancer metastasis [1]. PC carcinogenesis arises from androgen-dependent localized cancer and progresses to androgen-independent metastatic disease [2]. For PC patients diagnosed with androgen-dependent disease, androgen deprivation therapy (ADT) in combination with surgery, radiotherapy and chemotherapy have been successful in controlling tumor growth and expansion. However, the disease can progress to androgen-independent metastatic disease by developing ADT resistance at which point the patients face reduced chance of survival [1, 3].

Evidence suggests that stromal cells are key in promoting PC progression. This reactive stroma, termed the tumor microenvironment (TME), is composed of fibroblasts, myofibroblasts, immune cells, endothelial cells and CAFs, where this last one is the main TME component (40 to 50% of total cell population) [1, 4]. NF cells are responsible for maintaining homeostasis in the tissue. However, when these fibroblasts surround the TME, they become activated by tumor cell-secreted factors which re-educate the cells to acquire the CAF phenotype. CAFs are described as spindle-shaped cells that can be identified by the overexpression of several markers such as: α-smooth musle actin (α-SMA), fibroblast specific protein 1 (FSP-1) and fibroblast activation protein (FAP) [5, 6]. Several studies have determined that CAFs play a critical role in promoting PC progression by inducing tumor cell growth, invasion, epithelial-to-mesenchymal transition (EMT), ADT resistance, and enhanced tumor cell colonization [7–11].

The literature has mostly elucidated the role of CAFs related to the modification of tumor cells in the primary tumor and in metastatic locations. However, Duda *et al*. demonstrated that CAFs can migrate together with circulating tumor cells (CTCs) as circulating cell aggregates. This collective migration unit enhances tumor cell survival and colonization in distant organs [12]. Later, Ao *et al.* identified the presence of circulating CAFs in blood samples from cancer patients, with the number of CAFs correlating with disease progression in breast, prostate and colon cancer [13]. Importantly, these prior studies demonstrated the presence of CAFs in the circulation and the significant role of circulating stroma cells in promoting cancer progression, however, the specific function of CAFs in the bloodstream has not been elucidated yet.

During cancer metastasis, tumor cells invade surrounding tissues and cells enter the bloodstream to disseminate. When the tumor cells enter into the blood vessels, they experience fluid shear stress (FSS) from 160 s^-1^ to 900 s^-1^ in the venous and arterial circulation, respectively. During the transit of CTCs, they can experience FSS exceeding 3,000 dyn/cm^2^ in the turbulent flows in larger blood vessels, vessel bifurcations and close to the walls of the heart [14]. FSS is considered the main cause of tumor cell death in the circulation [15, 16]. Successful metastasis therefore depends on CTCs that somehow withstand the harsh shear stress environment to form secondary tumors in distant tissues. We hypothesize that CAFs confer resistance to high magnitude FSS to tumor cells in the circulation when the cells are incorporated into cell aggregates in collective migration units.

In the present study, using a 3D model, we determined that recently activated CAFs, termed reactive CAFs rather than differentiated CAFs, induced FSS resistance to PC cells by forming stable cell aggregates which can maintain their viability and proliferative capability. We also found that reactive CAF derived factors induce resistance to FSS to tumor cells but to a lesser degree than intercellular contact. Here we elucidate a cellular mechanism that explains, for the first time, the role of circulating CAF in the bloodstream by promoting CTC survival and migration.

## RESULTS

### Optimal experimental conditions to develop tumor cell and fibroblast co-culture in spheroid form

**Figure 1:**
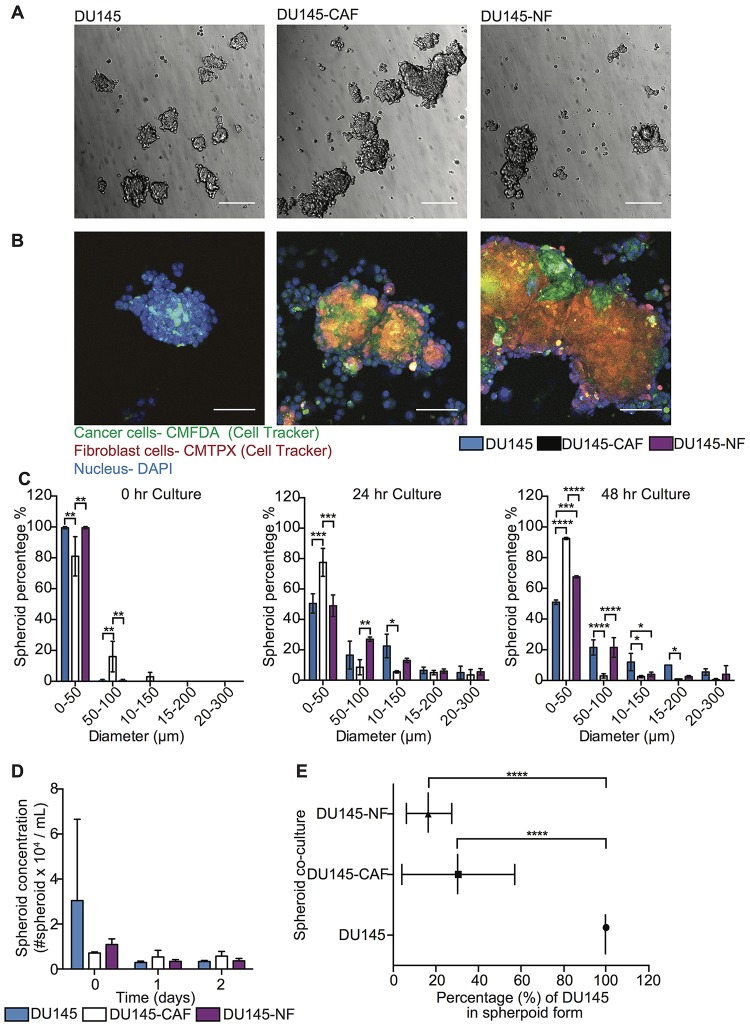
Characterization of DU145 and stromal cell co-culture in spheroid growth and composition. (**A**) Bright field images of DU145, DU145-CAF and DU145-NF spheroids after 24 hr in culture. Scale bar represents 200 μm. (**B**) Immunofluorescent staining of DU145, DU145-CAF and DU145-NF spheroids after 24 hr in culture (DU145 in green, fibroblast in red and nucleus in blue). Scale bar of 100 μm. (**C**) Bar chart represents the size distribution of DU145, DU145-CAF and DU145-NF spheroids at 0, 24, and 48 hr in culture (mean and range; *n =* 3 of two co-culture wells). Significance effect of DU145 and stromal cell co-culture (^*^ *P <* 0.0498, ^**^ *P* < 0.0067, ^***^ *P* < 0.0003 and ^****^ *P <* 0.0001) in the size distribution of spheroids at 0, 24 and 48 hr in culture was calculated using two-way ANOVA. (**D**) Bar graph represents the spheroid concentration of DU145, DU145-CAF and DU145-NF spheroids at 0, 24 and 48 hr of growth (mean and S. D.; *n =* 3 of two co-culture wells). Non-significance (*P <* 0.9997) was calculated using two-way ANOVA. (**E**) Scatter dot chart represents the composition of cells per spheroid in different co-culture conditions (mean and S. D.; *n* = 3 of two co-culture wells). Significance (^****^ *P* < 0.0001) was calculated via two-way ANOVA.

**Figure 2:**
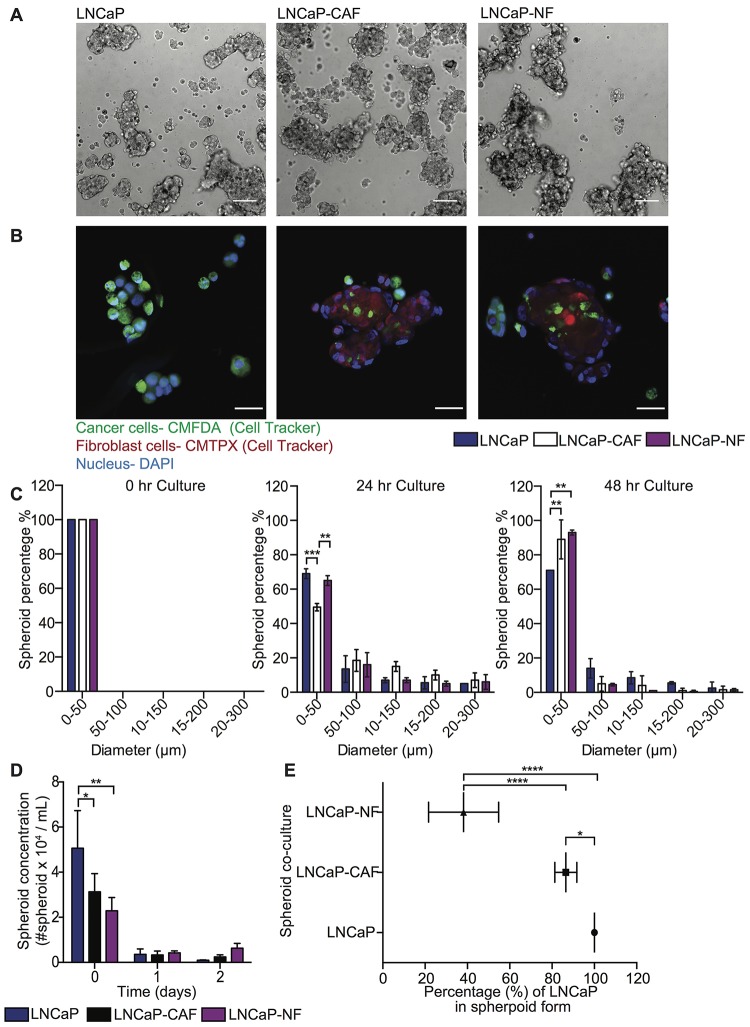
Characterization of LNCaP and stromal cell co-cultures in spheroid growth and composition. (**A**) Bright field images of LNCaP, LNCaP-CAF and LNCaP-NF spheroids after 24 hr in culture. Scale bar of 200 μm. (**B**) Immunofluorescent staining of LNCaP, LNCaP-CAF and LNCaP-NF spheroids after 24 hr in culture (LNCaP in green, fibroblast in red and nucleus in blue). Scale bar of 100 μm. The images were cropped to show the respective spheroid using a size of 270.79 μm × 270.79 μm. The brightness and contrast of the whole image were then adjusted to show a bright field signal that would sufficiently show in a printed version of the manuscript. The images were not modified by sections. (**C**) Bar chart represents the size distribution of LNCaP, LNCaP-CAF and LNCaP-NF spheroids at 0, 24, and 48 hr in culture (mean and range; *n* = 3 from two co-culture wells). Significance effect of LNCaP and stromal cell co-culture (^**^ *P* < 0.0041 and ^***^ *P* < 0.0006) in the size distribution of spheroids at 0, 24 and 48 hr in culture was calculated using two-way ANOVA. (**D**) Bar graph represents the spheroid concentration of LNCaP, LNCaP-CAF and LNCaP-NF spheroids at 0, 24 and 48 hr of growth (mean and S. D.; *n* = 3 from two co-culture wells). Significance (^*^ *P* = 0.0406 and ^**^ *P* = 0.0058) was calculated using two-way ANOVA. (**E**) Scatter dot chart represents the composition of cells per spheroid in different co-culture conditions (mean and S. D.; *n* = 3 from two co-culture wells). Significance (^*^ *P* = 0.0232 and ^****^ *P* < 0.0001) was calculated via two-way ANOVA.

To investigate the role of fibroblasts in inducing FSS resistance in metastatic prostate tumor cells, 3D mono- and co-culture of tumor and fibroblast cells was characterized to determine the optimal growth conditions by measuring the following parameters over time: (i) spheroid concentration, (ii) size distribution, and (iii) the incorporation of heterotypic cells in spheroids. PC cell lines DU145 and LNCaP were mono- and co-cultured with CAF and NF on PDMS coated plates for three days and bright field images acquired to monitor aggregate development over time (Figures 1A and 2A). Within a few hours of culture, less than 10% of cell aggregates were visible, and most cells had not formed spheroid structures yet. After one day of culture, cell aggregates developed into spheroids. However, after two days of culture the existing spheroids began to aggregate among themselves, forming larger networks that exhibited less spherical structure. Importantly, other existing spheroids showed deterioration at later stages, as determined by the increased presence of single cells. Overall, we found that 16–24 hr was the optimal incubation time to allow cancer cells and fibroblasts to form stable spheroids for further experiments (Figures 1C and 2C). However, the incorporation of cells during spheroid formation is dependent on cancer cell type. For DU145, 50% cells formed well-integrated DU145 mono-culture and DU145-NF co-culture spheroids, whereas only 30% of cells form stable DU145-CAF spheroids with a size range of 50-300 μm (Figure 1C). These DU145, DU145-CAF and DU145-NF spheroids have an estimated concentration of 2,927, 5,399 and 3,100 spheroids/mL, where only 30% and 20% of DU145 were incorporated in the CAF and NF co-culture spheroids (Figure 1B, 1D and 1E). However, in LNCaP spheroid formation, we observed that 50% of cells formed compact LNCaP-CAF spheroids, whereas less than 30% of cancer cells formed LNCaP mono-culture and LNCaP-NF co-culture spheroids (Figure 2C). These LNCaP, LNCaP-CAF and LNCaP-NF spheroid, showed an estimated concentration of 3,580, 3,330, 4,210 spheroid/ml, respectively, where 80% and 40% of LNCaP where incorporated in the CAF and NF co-culture spheroids, respectively (Figure 2B, 2D and 2E). Collectively, we found that the integration pattern of cells into the spheroid depends on the tumor cell type.

### Fibroblasts impact cancer cell viability at high FSS via direct intercellular contact

**Figure 3:**
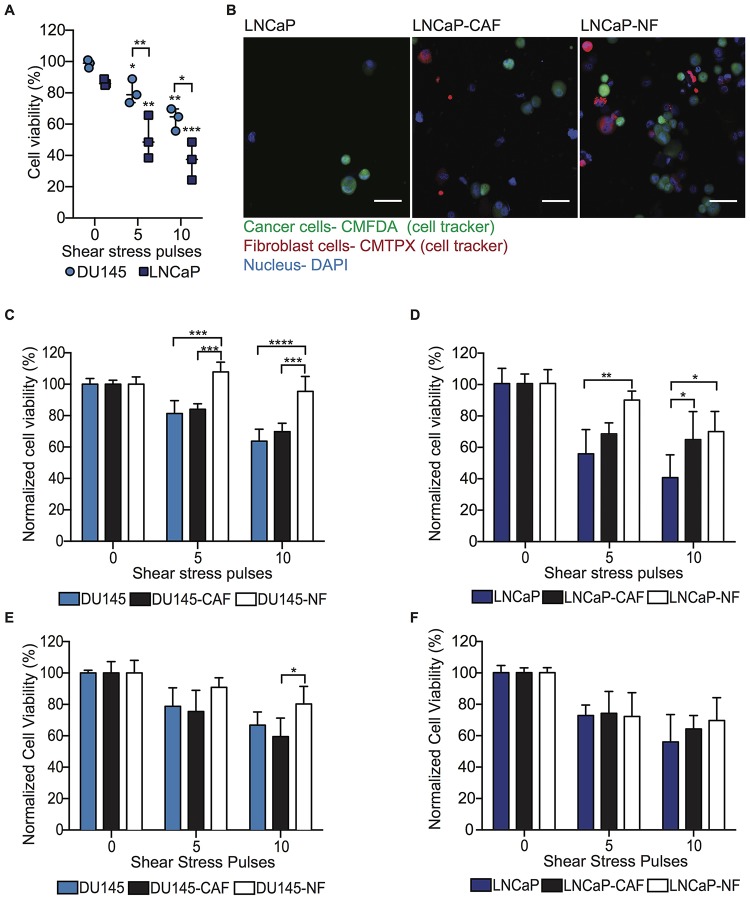
NF induces FSS resistance in metastatic PC cells through intercellular contacts and soluble derived factors. (**A**) Scatter dot chart represents the cell viability percentage for DU145 and LNCaP cells before and after being exposed to high magnitude FSS (mean and S. D.; *n* = 3). Significant (^**^ *P =* 0.0013, ^***^0.0005 and ^****^ *P <* 0.0001) reduction of cell viability was calculated using two-way ANOVA. (**B**) Immunofluorescent staining of LNCaP, LNCaP-CAF and LNCaP-NF spheroids after being exposed to high FSS (LNCaP in green, fibroblasts in red, and nucleus in blue). Scale bar of 50 μm. (**C**) Bar graph represents the normalized cell viability for DU145, DU145-CAF and DU145-NF spheroids at different magnitudes of FSS (mean and S. D.; *n* = 3). Significant effect of co-culture (^***^ *P* < 0.0004 and ^****^ *P* < 0.0001) inducing FSS resistance in DU145 was calculated using two-way ANOVA. (**D**) Bar graph shows the normalized cell viability for LNCaP, LNCaP-CAF and LNCaP-NF spheroids exposed to FSS (mean and S. D.; *n*=3). Significance (^*^ *P* = 0.0170 and ^**^ *P* = 0.0056) was calculated using two-way ANOVA. (**E**) Bar graph represents the normalized viability percentage of DU145 spheroid culture in conditioned media (mean and S. D.; *n =* 3). Significance effect of conditioned media (*P =* 0.0409) in the survival of DU145 cells was calculated using two-way ANOVA. (**F**) Bar chart represents normalized cell viability percentage of LNCaP spheroids culture in conditioned media (mean and S. D.; *n* = 3). Non-significance (*P =* 0.06913) was calculated using two-way ANOVA.

To investigate the role of CAF and NF in inducing resistance to high FSS in PC cells, DU145 and LNCaP mono-cultured and co-cultured with CAF and NF in spheroids were exposed to high magnitude FSS (5,920 dyn/cm^2^) and then cell viability assessed with flow cytometry to detect Hoechst-positive cells. Cells that lose viability due to the high FSS show the Hoechst dye exiting the permeable non-viable cells, preventing them from being counted. High FSS was found to reduce 20% and 50% cell viability in DU145 and LNCaP cells, respectively. Increasing the duration of high FSS exposure further decreased cell viability to 40% and 60% in corresponding cell lines (Figure 3A). However, DU145 cells co-cultured with NFs exhibited significantly higher cell viability compared to DU145 mono-culture or DU145 co-cultured with CAFs at high magnitude of FSS (Figure 3B and 3C). Similarly, LNCaP cells co-cultured with NF showed slightly higher cell viability compared with LNCaP and LNCaP-CAF (Figure 3D). To validate that the cytoprotective effect of NF in PC cells is independent of the cancer cell phenotype, PC3 cells were mono-cultured and co-cultured with NF and CAF in spheroid form using the same experimental conditions. FSS reduced 15% of cell viability and increasing the duration of FSS further decreased the cell viability to 20 and 35% (Supplementary Figure 1A). When these spheroids were exposed to higher magnitude of FSS, PC3-NF showed higher cell viability than PC3 mono-culture and PC3-CAF spheroids (Supplementary Figure 1B). These findings indicate that NFs promote cell survival in high FSS, which CAFs do as well, albeit to a lesser degree. These results indicate that fibroblasts may promote cancer cell metastasis by serving a cytoprotective function against high FSS. The next question to address was whether this cytoprotective function of fibroblasts is via direct intercellular contact or via fibroblast-secreted factors.

### Fibroblast-derived factors impact cell viability after high FSS in tumor cells experiencing bloodborne metastasis-like conditions

To determine the role of fibroblast-derived factors in inducing a resistance response of tumor cells to FSS, DU145 and LNCaP spheroids were cultured using CAF- and NF-conditioned media and exposed to high magnitude FSS under the same experimental conditions. DU145 spheroids cultured with NF-conditioned media showed higher cell viability compared with DU145 spheroids and DU145 spheroid culture with CAF conditioned media. This increase in cell viability was observed in cells subjected to high FSS for an extended time compared to shorter time exposure (Figure 3E). Similar cytoprotective effect was observed in PC3 spheroids as well (Supplementary Figure 1C). However, LNCaP spheroids did not show an increase in cell viability due to the NF conditioned media as we observed in the other two androgen-independent cell lines (Figure 3F). To identify the soluble factors that play a role in conferring resistance to shear forces, a cytokine array was performed and the level of expression of these factors in the NF and CAF conditioned media were determined and displayed in Table 1. Collectively, these findings suggest that fibroblast-derived factors may confer resistance to FSS to DU145, PC3 but not LNCaP. Thus, the cytoprotective role of fibroblasts may be a synergy of intercellular contact and fibroblast-derived factors that have a strong effect that is androgen-independent cell type specific. To this point, we have found that a function of NF is to promote the survival of tumor cells in high FSS. However, the proliferative capability of the tumor cells should be determined to confirm the impact of the NF role in promoting the formation of metastases.

**Table 1:** Identification and enumeration of cytokines released by NF and CAF in media under regular culture conditions

Cytokines	NF (fold concentration compared to CAF)	CAF
Interleukin (IL)-1α, 2, 3, 4, 5, 10, 12, 13	1	1
IL-1β	1.3	1
IL-6	0.3	1
IL-7	0.6	1
IL-8	0.5	1
IL-15	4	1
Monocyte chemoattractant protein (MCP) 1 and 3	5	1
MCP-2	1.5	1
Tumor necrosis factor (TNF)-α	1	1
TNF-β	1.3	1
Epidermal growth factor	0.5	1
Macrophage and granulocyte colony-stimulating factor	1	1
Insulin-like growth factor 1	1	1
Epithelial neutrophil-activating protein 78	5	1
CC chemokine (CCL1)	1.4	1
CCL (2, 5, 17)	1	1
Angiogenin	1	1
Monokine induced by gamma interferon	1	1
Oncostatin M	1	1
Macrophage inflammatory protein 1	3	1
Thrombopoietin	1.3	1
Growth-regulated oncogene (GRO, GRO-α)	0.2	1
Vascular endothelial growth factor (VEGF)	1	1
Stem cell factor	1.3	1
Platelet-derived growth factor (PDGF BB)	1	1
Stromal cell-derived factor 1 (SDF-1)	1.5	1
Leptin	1	1
Interferon gamma (IFNγ)	0.7	1

### Fibroblasts preserve the proliferative ability of PC cells during high FSS

**Figure 4:**
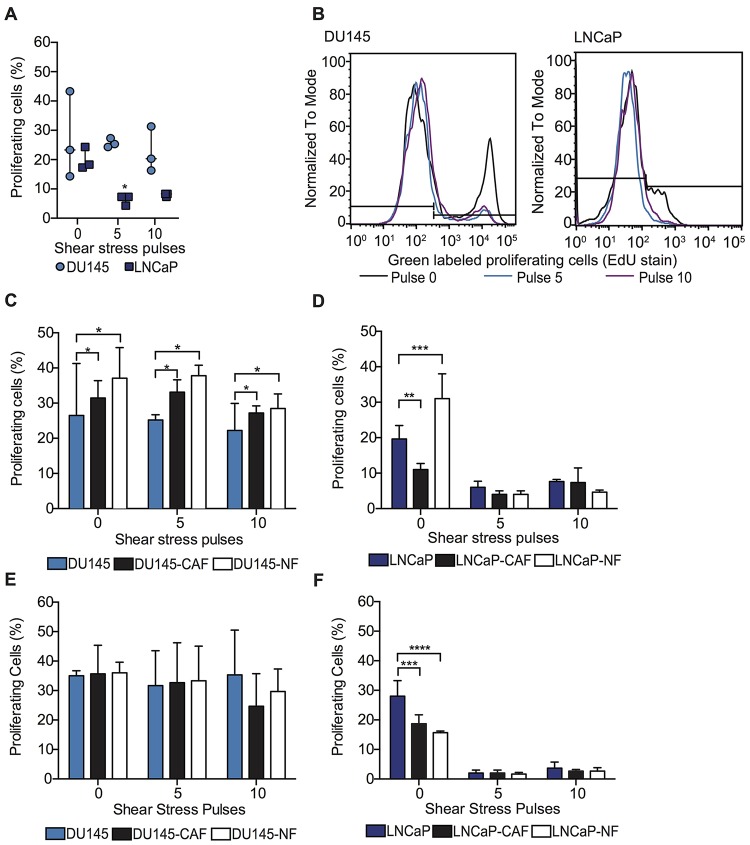
Fibroblasts maintain the proliferative ability of PC cells within high FSS through intercellular contact with PC cells. (**A**) Scatter dot chart represents the percentage of DU145 and LNCaP mono-culture spheroids in the proliferating stage (S) of the cell cycle before and after undergoing high FSS (mean and S. D.; *n* = 3). Significant reduction of cell proliferation (^**^ *P* < 0.0035) calculated using two-way ANOVA. (**B**) Histogram represents the intensity of proliferating tumor cells (DU145 and LNCaP) before and after being subjected to FSS. Black curve represents the proliferating cells under static conditions, while blue and violet curves represent the proliferating tumor cells after 5 and 10 shear pulses. (**C**) Bar graph represents the percentage of proliferating DU145 cells (positive EdU stained cells) before and after being exposed to high magnitude FSS (mean and S. D.; *n* = 3). Significant effect of co-culture (^*^ *P* = 0.0226) in DU145 proliferation. (**D**) Bar graph shows the percentage of proliferating cells before and after being exposed to high magnitude FSS (mean and S. D.; *n* = 3). Significant increase in LNCaP proliferation (^**^ *P =* 0.0091, ^***^ *P =* 0.0009 and ^****^ *P <* 0.0001) when co-cultured with CAF and NF under static conditions. (**E**) Bar graph of the percentage of proliferating DU145 cells after being subjected to high FSS (mean and S. D.; *n =* 3). Non-significance (*P =* 0.8268) was calculated via two-way ANOVA. (**F**) Bar chart represents the percentage of proliferating LNCaP cells after being exposed to high FSS (mean and S. D.; *n =* 3). Significant effect (*P <* 0.0001) of conditioned media in LNCaP proliferation under static conditions.

Beyond viability, the impact of co-culture with fibroblasts on cancer cell proliferation was also assessed. In this study, we found that high FSS did not affect the proliferation of viable DU145 spheroids. However, FSS reduced the proliferation of viable LNCaP spheroids fourfold compared to LNCaP spheroids under static conditions (Figure 4A and 4B). Regarding PC3 spheroids, FSS reduced 10% of proliferation of viable PC3 spheroids compared with spheroids at static conditions (Supplementary Figure 2A and 2B). Importantly, DU145 co-cultured with CAF or NF in spheroids showed an increase of proliferating cells compared to DU145 spheroids before and after being exposed to high magnitude FSS (Figure 4C). The opposite effect was observed in LNCaP spheroids, where LNCaP co-culture with NF caused an increase in cell proliferation under static conditions but no such effect observed for CAF culture. After the LNCaP mono-culture and co-culture spheroids were exposed to high FSS, the cell proliferation of LNCaP was dramatically reduced for all different culture conditions (Figure 4D). These observations were also found in PC3 spheroids (Supplementary Figure 2C). Collectively, this indicates that NFs enhance cancer cell proliferation under static conditions, indicating a proliferative role in tumor progression for DU145, LNCaP and PC3. Also for DU145 cells, NFs and CAFs were found to promote cancer cell proliferation after high-FSS exposure, suggesting that fibroblasts could play a role in forming secondary tumors by protecting cancer cell proliferative capacity from FSS in cells that experience bloodborne but not lymphatic transit.

### Fibroblast-derived factors do not impact the proliferation of PC cells under high FSS

To investigate if the variation in proliferation rate of tumor cells after being exposed to FSS is related to intercellular contact or fibroblast-derived factors, DU145, LNCaP and PC3 spheroids were cultured with CAF- and NF- conditioned media. After the spheroids were exposed to FSS, it was found that the CAF and NF conditioned media did not increase tumor cell proliferation in either DU145, LNCaP and PC3 cells (Figure 4E and 4F). However, PC3 co-cultured with NF conditioned media in spheroid form slightly enhanced the proliferation of PC3 under static conditions (Supplementary Figure 2D). Taken together, the dominant factor maintaining the proliferative capacity of tumor cells under FSS is thus the cellular cluster of tumor cells with CAF and NF. In this study, we found that NF proved to strongly impact tumor cell viability in comparison to CAFs, which is an unexpected finding that contradicts our previous hypothesis. Therefore, the next question to address was if the 3D co-culture condition induces a spontaneous activation of NF into CAF.

### Cancer cells re-educate fibroblasts to exhibit a CAF-like phenotype

**Figure 5:**
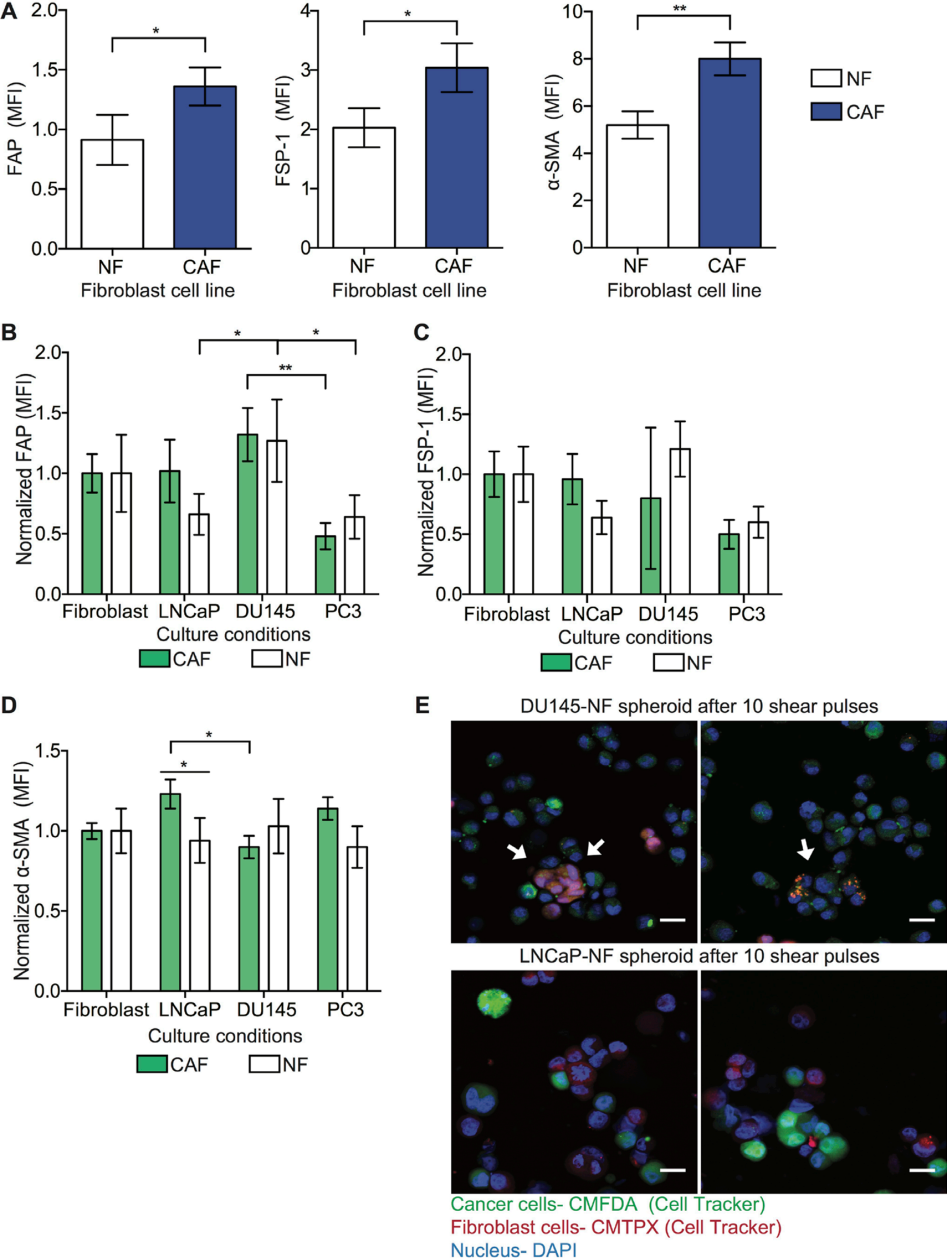
NFs are activated into CAF phenotype. (**A**) Bar graph shows the mean fluorescence intensity (MFI) of FAP, FSP-1 and α-SMA expression in NF and CAF cell lines in monoculture conditions (mean and S. D.; *n* = 3). Significant increase (^*^ *P =* 0.0419 and ^**^ *P <* 0.0293) in expression of these biomarkers in CAF compared to NF. (**B**) Bar graph represents the normalized MFI of FAP expression in NF and CAF co-cultured with cancer cells in spheroid form (mean and S. D.; *n* = 3). Significance effect of co-culture (^*^ *P* < 0.0252 and ^**^ *P* = 0.0022) in FAP expression in NF was calculated using two-way ANOVA. (**C**) Bar graph represents the normalized MFI of FSP-1 expression in NF and CAF co-culture with cancer cells in spheroid form (mean and S. D.; *n* = 3). Non-significant fluctuation in FSP-1 expression in NF compared to CAF (*P =* 0.6727) was estimated by using two-way ANOVA. (**D**) Bar graph represents the normalized MFI of α-SMA expression in NF and CAF co-culture with cancer cells (LNCaP, DU145 and PC3) in spheroid form (mean and S. D.; *n* = 3). Significant change in α-SMA expression (^*^ *P* < 0.0276) was calculated using two-way ANOVA. (**E**) Immunofluorescence staining of LNCaP-NF and DU145-NF spheroids after being exposed to high magnitude FSS (LNCaP in green, NF marker in red and nucleus in blue). Scale bar is 20 μm.

To determine if tumor cells can activate NF into a CAF-like cellular state, CAF and NF were cultured in mono-culture and in co-culture with tumor cells (DU145, LNCaP and PC3) and the variation of CAF marker (FAP, FSP-1 and α-SMA) expression was evaluated. In this study, we validated the cellular phenotype by measuring the level of CAF markers in NF and CAF cell lines in spheroid form. Here we found that the CAF cell line showed high expression of FAP, FSP-1 and α-SMA markers compared to the NF cell line (Figure 5A). However, when the NF were co-cultured with tumor cells, these co-cultured spheroids showed altered level of α-SMA and FAP but not FSP-1 (Figure 5B–5D). When the NF is co-cultured with DU145 in spheroid form, these showed higher FAP and α-SMA levels in comparison to the monoculture conditions (Figure 5B and 5D). To determine that the spheroid culture conditions do not induce NF activation, the α-SMA expression was evaluated in CAF and NF culture in 2D and 3D form. We found that 3D culture of fibroblasts does not change the expression of α-SMA marker compared with 2D culture (Supplementary Figure 3A). We questioned whether the spontaneous activation of NF cells in the co-cultured spheroids with DU145 and LNCaP is due to intercellular communication by exosomes or macrovesicles. Therefore, we imaged the NF and CAF co-culture with DU145 and LNCaP after being exposed to high magnitude FSS. Here we found in the confocal images that the cytoplasm of NF and CAF contains particles labeled with Cell Tracker corresponding to tumor cells (Figure 5E). Together, we found that when the cells are in the spheroid form however, the tumor cells may communicate with fibroblasts via exosomes which induce the activation of NF to a CAF cellular state. Thus, the fibroblast response to cellular signals depends on the cancer cell type.

## DISCUSSION

CAFs have become recognized as a critical player during cancer progression by regulating tumor cell proliferation, invasion and formation of metastases [5]. Recently, studies have revealed the presence of CAFs in the circulation of cancer patients, and their level in blood biopsy correlates with cancer progression and worse prognosis [13]. In this study, for the first time we demonstrated the significant role of CAFs in the circulation by promoting the survival of tumor cells in FSS. We found that reactive CAFs induce resistance to FSS to tumor cells by forming a protective cellular nodule and by soluble factors such as: CCL2, CCL7 and CXCL5. Importantly, reactive CAFs maintain the proliferative capacity of tumor cells in cellular aggregates within a high magnitude of hemodynamic forces. The cytoprotective role of reactive CAFs was strongly observed within PC cells that carry out bloodborne rather than lymphatic metastatic progression, establishing that the function of CAFs is specific to cancer cell type.

Regarding the literature, CAFs are not a type of cell but rather a cellular state. In other words, CAFs represent a heterogeneous population of cells, which trigger different cellular responses. In this study, we determined that NF are spontaneously activated to become α-SMA positive myofibroblasts by gaining a CAF phenotype. The NF differentiation into CAF might be attributed to spontaneous activation due to the intercellular interactions with tumor cells [17, 18]. However, Mellone M. *et al.* demonstrated that senescence of NF promotes their differentiation into α-SMA positive myofibroblasts [19]. Previous studies suggest that the CAF cell population induced by senescence of NF may promote EMT, proliferation and invasiveness in prostate cancer [20].

The reactive CAF population that we determined to be a dominant factor in inducing FSS resistance in tumor cells expresses higher ?-SMA and FAP compared to differentiated CAF. Several studies have reported that fluctuation in ?-SMA expression level in CAFs can differentiate these in two different cellular states: reactive state (higher α-SMA and FAP compared to differentiated CAF. Several studies have reported that fluctuation in α-SMA expression level in CAFs can differentiate these in two different cellular states: reactive state (higher α-SMA) and quiescent state (lower α-SMA) [21, 22]. Importantly, studies have reported that higher α-SMA expression in stromal cells correlates with tumor aggressiveness, progression, and worse prognosis [23, 24]. We thus conclude that in spheroids, reactive CAFs and cancer cells interact by forming strong cellular adhesions, which correlates with high cell viability and stable proliferative capability within high FSS environment. This implies that the mechanism in which CAFs protect tumor cells from FSS involves forming stable cell aggregates that can persist even when subjected to FSS over 1,000 dyn/cm^2^ along with the secretion of soluble factors such as CCL2, CCL7 and CXCL5. Based on the literature, these soluble factors activate nuclear factor-κB (NF-κB) in prostate cancer cells in which the downstream effect is associated with the enhancement of cell survival, invasion and EMT in cancer cells [25–27]. As result of our study and the current literature evidence, we suggest that reactive CAFs confer resistance to tumor cells via NF-κB signaling pathway. However, further studies should be done to fully elucidate the signaling pathway involved in the survival of tumor cells under FSS.

During metastatic progression, other host cells were found to have an important function by promoting the survival of tumor cells in the bloodstream. A body of evidence has well established the role of platelets in tumor cell survival by creating a protective shield surrounding tumor cells via fibrinogen interactions. Studies have revealed the prominent role of platelets in promoting tumor cell survival and arrest in distant organs [28–30]. In this study, we demonstrated that platelets are not the only mechanism that tumor cells may use to improve their survival in the circulation. We found that reactive CAF not only impact cell viability, as platelets, but also maintain the proliferative capability of tumor cells within FSS, which is crucial to bypass the colonization period and, eventually, enhance the overgrowth in distant organs. Thus, high levels of CAF-CTC aggregates in biopsy from cancer patients should be considered an important marker to potentially predict the clinical outcome.

In conclusion, we have demonstrated that reactive CAFs confer FSS resistance to prostate tumor cells in cellular aggregates via intercellular contacts as well as soluble derived factors. Importantly, this heterotypic cellular cluster can maintain the proliferative capability of prostate tumor cells within FSS. Indeed, reactive CAF cells are a key player in promoting the survival of tumor cells in the circulation, which suggests the importance of circulating CAF in the bloodstream as a biomarker for worse prognosis in metastatic disease as well as a promising target for novel cancer therapeutics.

## MATERIALS AND METHODS

### Cell culture

In this study, the following cells were utilized: DU145, LNCaP, WPMI-1 (NF) and hTERT PF179T CAF (CAF). DU145 represents an androgen-independent PC cell derived from a metastatic location in the brain. LNCaP is an androgen-dependent PC cell derived from a metastatic location in the lymph node. Both cell lines were used to explore different aspects of metastatic PC such as androgen dependency and metastatic pathway (bloodborne vs lymphatic metastasis). WPMY-1 represents a myofibroblast stromal cell derived from the peripheral zone of the normal prostate while hTERT PF179T CAF represents a prostate fibroblast cell derived from the prostate cancer stroma [31, 32]. All the cell lines were obtained from ATCC and cultured according to the manufacturer protocols using the following media: EMEM (Gibco), F-12K (Gibco) and DMEM (Gibco). All cell lines were cultured at 37 °C with 5% CO_2_.

### Spheroid formation

Single cell suspensions were recovered from culture flasks using 0.25% trypsin solution (Gibco) for 10 min. For the characterization of spheroid growth, the cancer cells were stained using Cell Tracker dye (green CMFDA dye, Invitrogen) and the fibroblasts were stained with red Cell Tracker dye (deep red dye, Invitrogen) using a working concentration of 25 μg/ml for 30 min in an incubator. For samples that were subjected to FSS, cancer cells were stained with 1 μg/ml of Hoechst stain (ThermoFisher) for 30 min in an incubator. Approximately, 50,000 cancer cells (DU145 or LNCaP) were mono-cultured and co-cultured with 50,000 CAF or NF cells in PDMS coated 24-well plates using 1 ml of media [33, 34]. The culture plates were placed in the incubator for 24 hr at 37 °C with 5% CO_2_.

### Characterization of spheroid growth

The mono-culture and co-culture spheroids were grown for three days. Bright field images were acquired using an Olympus IX81 motorized inverted microscope. The enumeration and diameter of spheroids formed per day were evaluated using Image J software. Regarding the composition of spheroids, confocal images were taken from mono-culture and co-culture spheroids using an LSM 710 Meta inverted confocal microscope. Using Image J software, the tumor and fibroblast cells were enumerated using the following criteria: cancer cells were identified as cells staining for green Cell Tracker and positive for nuclear staining via DAPI, and fibroblasts were identified as cells staining for red Cell Tracker and positive for nuclear staining via DAPI.

### Shear of mono and co-culture spheroids at high magnitude FSS

Spheroid suspensions were lifted from 24-well plates and placed in 5 mL syringes of gauge 30 (BD). Each sample (mono-culture and co-culture) was then exposed to 5,920 dyn/cm^2^ for 1.08 ms using a syringe pump (Harvard Apparatus, Holliston MA) and were allowed to rest for 2 min between each shear condition to mimic the time it takes a cell to circulate through the body [15]. Spheroids were exposed to five or ten shear pulses. The cells were then plated with regular media overnight. After 24 hr, cell viability was determined by enumerating the viable cancer cell percentage (fraction of cancer cells retaining Cell Tracker dye) using flow cytometry.

### Cancer cells cultured with fibroblast-derived conditioned media

CAF and NF cells were cultured for weeks while the conditioned media was collected and stored at 4°C. Cancer cells were then cultured in mono-culture spheroids as described above, using conditioned media in place of regular culture media.

### Measurement of fibroblast-derived cytokines

500,000 fibroblast cells (NF and CAF) were cultured in 6-well plates using 2 mL of culture media. The culture plates were placed in the incubator for 24 hr at 37 ^°^C with 5% CO_2_. Then, the media was replaced with serum free media, to avoid false positives. After 24 hr, the media was removed from the culture plates and placed in microtubes. These samples were spun down at 5,000 RPM for 5 min to remove the cellular debris. The media was incubated with a membrane that contains antibodies able to identify 42 cytokines following the manufacturer protocol (Abcam, human cytokine array membrane). At the end, the membrane was imaged using an ImageQuant LAS-4000 system (GE Healthcare). The mean pixel intensity was determined using Image J software.

### Cell proliferation assay using EdU staining

After the cells were sheared, they were cultured in multi-well plates using supplemented media overnight. One day later, 10 μM of EdU (Click-iT EdU flow cytometry cell proliferation assay, ThermoFisher) was added to each well and incubated for 2 h at 37 °C with 5% CO_2_. The cells were then lifted from the culture plates and the cell suspensions prepared using 0.25% trypsin for 10 min. The cells were then fixed and permeabilized using 100 μl of Click-iT fixative and 1× Click-iT saponin-based solution following the instructions of the manufacturer (Click-iT EdU flow cytometry cell proliferation assay, ThermoFisher). The cells were then stained using Click-iT EdU reaction cocktail for 30 min and the percentage of proliferating cells evaluated using a flow cytometer [35].

### α-SMA and vimentin expression in CAF and NF

CAF and NF cells were mono-cultured and co-cultured with DU145 and LNCaP for 24 h under spheroid conditions as described above. The cell suspensions were then prepared using 0.25% trypsin for 10 min. The cells were washed and fixed with 1% BSA (Sigma) for 5 min and 4% Paraformaldehyde (Electron Microscopy Sciences) for 15 min at room temperature. The cells were then permeabilized using 0.25% triton (Sigma-Aldrich) for 15 min and incubated with 10 μg/ml of mouse anti-α-SMA conjugated eFluar 660 (eBiocience), anti-vimentin conjugated Alexa Fluor 488 (Biolegend) and 1% BSA for 30 min. Cell suspensions were washed three times with 1% BSA after immunostaining. α-SMA and vimentin expression was evaluated in fluorescently labeled fibroblast cells using flow cytometry.

### Statistical analysis

All statistical analyses were carried out using PRISM 6.0 for Mac OS X. The figure legends contain the detailed information about the number of samples, the technical repeats and the statistical test used for each respective experiment. ANOVA tests were used to compare more than two groups. For multiple comparisons using ANOVA test, Turkey adjustments were applied and the adjusted *P* value used. All of the statistical tests were treated as two-sided and calculated at a level of significance of alpha = 0.05. For flow cytometry, FlowJo software 10 was used to perform all data analysis.

## SUPPLEMENTARY MATERIALS


