# Supplementary material for: Cancer associated fibroblasts confer shear resistance to circulating tumor cells during prostate cancer metastatic progression
Source: Oncotarget. 2020 Mar 24;11(12):1037–50. doi: 10.18632/oncotarget.27510 (PMC7105166; doi:10.18632/oncotarget.27510)
Supplement: Supplementary file 1 [file oncotarget-11-1037-s001.pdf]

Cancer associated fibroblasts confer shear resistance to circulating tumor cells during prostate cancer metastatic progression

SUPPLEMENTARY MATERIALS

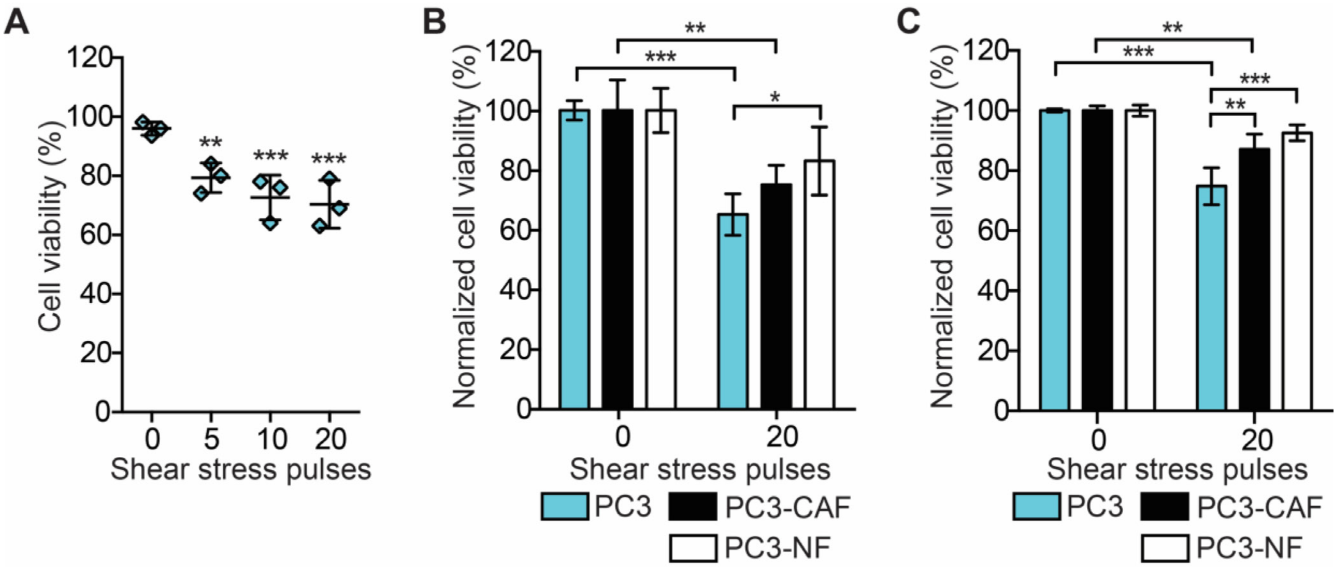

Supplementary Figure 1: NF-induces FSS resistance in metastatic androgen independent PC3 cells through intercellular contacts and soluble derived factors. (**A**) Scatter dot chart represents the cell viability percentage for PC3 cells before and after being exposed to high magnitude FSS across three different shear stress pulses (mean and S.D.; $n = 3$). Significant ($^{*}P = 0.0315$, $^{**}P = 0.0051$ and $^{***}P = 0.0005$) reduction of cell viability was calculated using one-way ANOVA. (**B**) Bar graph represents the normalized cell viability for PC3, PC3-CAF and PC3-NF spheroids at high magnitude of FSS (mean and S.D.; $n = 3$). Significant effect of co-culture ($^{*}P = 0.0454$, $^{**}P = 0.0078$ and $^{***}P = 0.0006$) inducing FSS resistance in PC3 was calculated using two-way ANOVA. (**C**) Bar graph represents the normalized viability percentage of PC3 spheroid culture in CAF and NF conditioned media (mean and S.D.; $n = 3$). Significance effect of conditioned media ($^{**}P < 0.0028$, $^{***}P = 0.0001$ and $^{****}P < 0.0001$) in the survival of PC3 cells was calculated using two-way ANOVA.

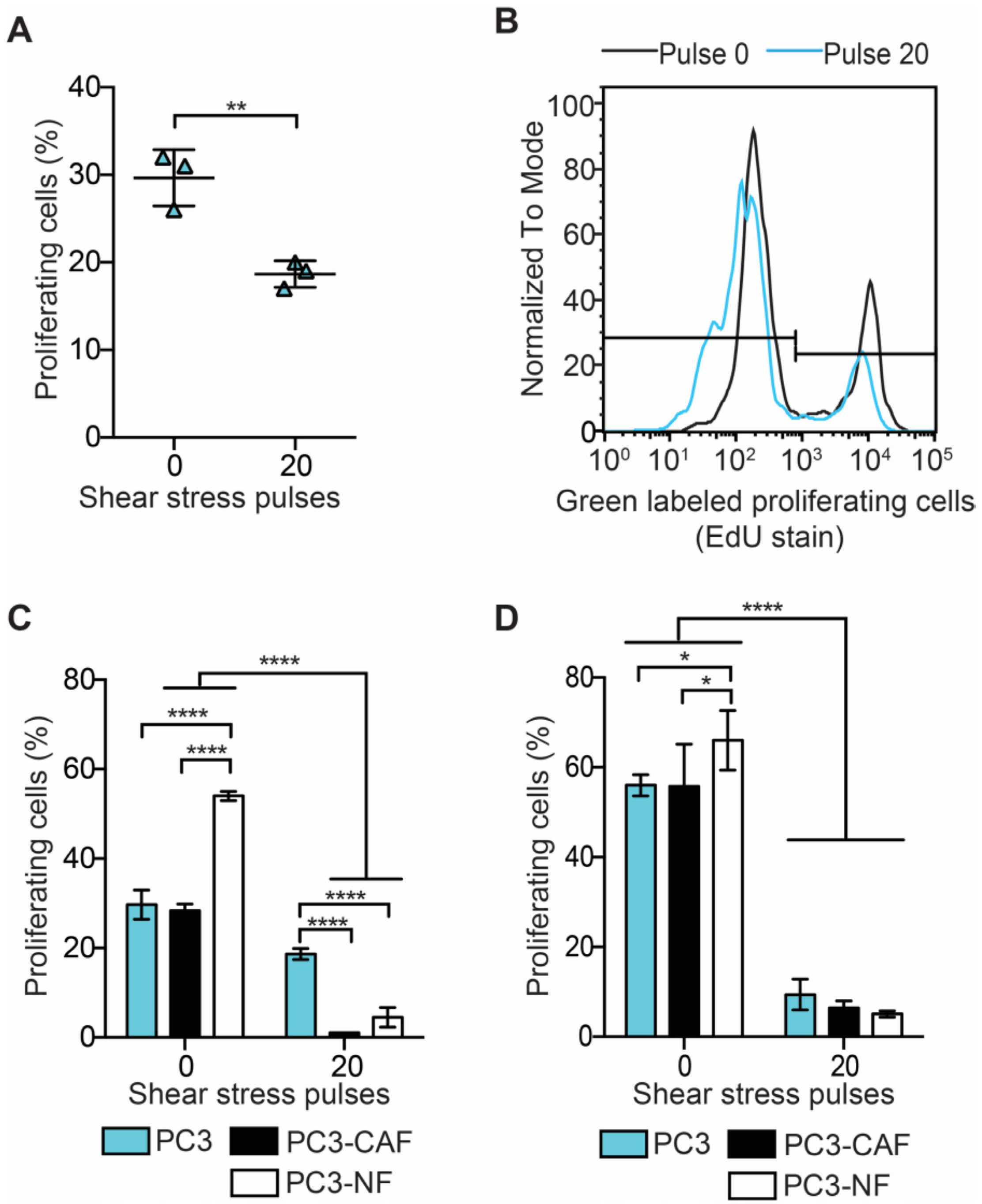

Supplementary Figure 2: Fibroblasts maintain the proliferative capability of androgen independent PC3 cells within high FSS through intercellular contact with PC cells. (**A**) Scatter dot chart represents the percentage of PC3 mono-culture spheroids in the proliferating stage (S) of the cell cycle before and after undergoing high FSS (mean and S.D.; $n = 3$). Significant reduction of cell proliferation ($^{**}P = 0.0059$) calculated using a unpaired t test. (**B**) Histogram represents the intensity of proliferating PC3 cancer cells before and after being subjected to FSS. Black curve represents the proliferating cells under static conditions, while light blue curve represents the proliferating tumor cells after 20 shear pulses. (**C**) Bar graph represents the percentage of proliferating PC3 cell co-culture with CAF and NF in spheroid form before and after being exposed to high magnitude FSS (mean and S.D.; $n = 3$). Significance effect of co-culture ($^{****}P < 0.0001$) in PC3 proliferation under static conditions. (**D**) Bar graph shows the percentage of proliferating PC3 spheroid culture with CAF and NF conditioned media before and after being exposed to high magnitude FSS (mean and S.D.; $n = 3$). Significant increase in PC3 proliferation ($^{*}P < 0.0299$ and $^{****}P < 0.0001$) when co-cultured with CAF and NF under static conditions was calculated using a two-ways ANOVA.

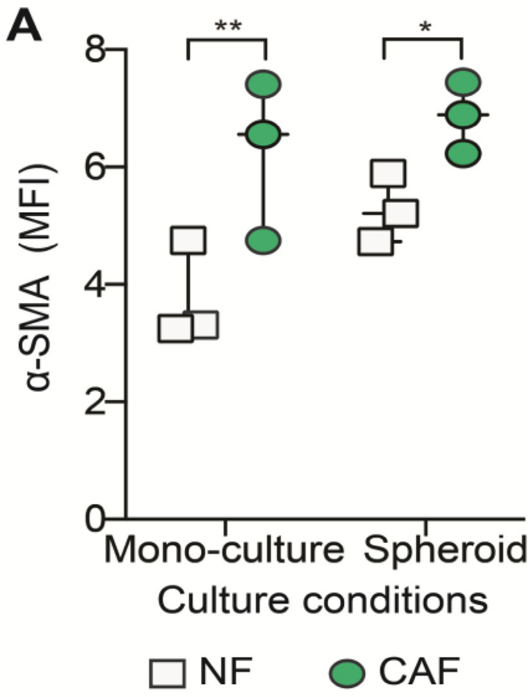

Supplementary Figure 3: Spheroid culture condition doesn't induce activation of NF into CAF. (**A**) Scatter dot plot shows the mean fluorescence intensity (MFI) of α-SMA expression in NF and CAF cell lines under two different culture conditions: mono-culture (two-dimensional culture) and spheroid (three-dimensional culture) (mean and S.D.; $n = 3$). Significance of α-SMA expression ($^{*}P = 0.0143$ and $^{**}P = 0.0050$) in CAF compared to NF under mono-culture conditions was computed using two-way ANOVA.
